# Women’s knowledge of symptoms of obstetric fistula, experiences, and associated factors in Sierra Leone

**DOI:** 10.1371/journal.pone.0312019

**Published:** 2024-10-24

**Authors:** Augustus Osborne, Peter Bai James, Camilla Bangura

**Affiliations:** 1 Department of Biological Sciences, School of Basic Sciences, Njala University, PMB, Freetown, Sierra Leone; 2 National Centre for Naturopathic Medicine, Faculty of Health, Southern Cross University, Lismore, Australia; 3 Faculty of Pharmaceutical Sciences, College of Medicine and Allied Health Sciences, University of Sierra Leone, Freetown, Sierra Leone; Harvard University T H Chan School of Public Health, ETHIOPIA

## Abstract

**Background:**

Obstetric fistula is a devastating childbirth condition that results from prolonged obstructed labour without timely medical intervention, leading to a tear between the birth canal and the bladder or rectum. It is a public health issue, particularly in low-income countries with limited access to quality maternal healthcare. This study aims to assess knowledge of fistula symptoms among women who had a fistula and its associated factors in Sierra Leone.

**Methods:**

Cross-sectional data from the 2019 Sierra Leone Demographic and Health Survey was used for the study. Our study comprised 15,574 reproductive women aged 15 to 49. Using a four-modelled approach, a mixed-effect multilevel binary logistic regression analysis was performed to assess the factors associated with knowledge of fistula symptoms among women who had a fistula. The results were presented as adjusted odds ratios with a 95% confidence interval.

**Results:**

The proportion of knowledge of fistula symptoms among women who had a fistula was 57.5% [55.3,59.7] in Sierra Leone. Women aged 20–49, particularly those between 40–44 [aOR = 2.82; 95% CI: 2.13, 3.73], were more likely to be aware of knowledge of fistula symptoms among women who had a fistula compared to teenagers (15–19). Women with higher levels of education [aOR = 2.07; 95% CI: 1.49, 2.88] were more likely to be aware of knowledge of fistula symptoms among women who had a fistula than those without education. Working women [aOR = 1.33; 95% CI: 1.14, 1.56], those who listened to the radio [aOR = 1.47; 95% CI: 1.30, 1.67] or used the internet [aOR = 1.64; 95% CI: 1.32, 2.05], and those with a high number of lifetime births [aOR = 2.00; 95% CI: 1.57, 2.54] were more likely to be aware of knowledge of fistula symptoms among women who had a fistula. Women who had ever had sex [aOR = 2.19; 95% CI: 1.73, 2.77], were pregnant [aOR = 1.37; 95% CI: 1.13, 1.66] or had terminated a pregnancy [aOR = 1.30; 95% CI: 1.07, 1.59] were more likely to be aware of knowledge of fistula symptoms among women who had a fistula. A female household head [aOR = 1.20; 95% CI: 1.05, 1.38] was associated with a higher likelihood of knowledge of fistula symptoms among women who had a fistula. On the other hand, larger household sizes [aOR = 0.86; 95% CI: 0.75, 0.97] and living in the Western region [aOR = 0.48; 95% CI: 0.31, 0.75] were associated with a lower likelihood of knowledge of fistula symptoms among women who had a fistula.

**Conclusion:**

Most reproductive-aged women in Sierra Leone have knowledge of fistula symptoms among women who had a fistula. Factors such as age, education, occupation, media exposure, parity, sexual activity, pregnancy status, abortion history, ethnicity, household structure, and geographic location influence the knowledge of fistula symptoms among women who had a fistula. Based on these findings, the government and partner organisations in Sierra Leone should implement comprehensive health education programs targeting reproductive-aged women, with a specific focus on obstetric fistula prevention, symptoms, and available treatment options.

## Introduction

Obstetric fistula (OBF) is a devastating childbirth injury that occurs when the soft tissue between the vagina, bladder, or rectum develops a hole due to prolonged and obstructed labour without timely medical intervention [1]. This condition results in continuous, involuntary leakage of urine, faeces, or both, causing severe physical and psychological suffering for affected women [1]. Obstetric fistula is primarily caused by obstructed labour, often resulting from factors such as early marriage, teenage pregnancy, inadequate antenatal care, and lack of access to skilled birth attendants [2, 3]. The symptoms of obstetric fistula include constant leakage of urine or faeces, foul odour, skin irritation, and recurrent infections [4]. The physical and emotional toll of this condition is immense, leading to social isolation, economic hardship, and, often, abandonment by partners and communities [4].

Globally, it is estimated that millions of women are living with obstetric fistula, with the highest prevalence observed in sub-Saharan Africa [5]. In this region, factors such as poverty, inadequate healthcare systems, and cultural practices contribute significantly to the incidence of this condition [6–12]. Sierra Leone has one of the highest rates of obstetric fistula in the world; while precise figures are limited due to underreporting, it is estimated that thousands of women in Sierra Leone are living with the condition [13], exacerbated by years of civil conflict, poor maternal health services, and limited access to education for women [13]. These factors have created a perfect storm for the perpetuation of obstetric fistula, making it a critical public health issue that demands urgent attention.

Sierra Leone has made strides in addressing obstetric fistula through various policies, programs, and activities. In collaboration with international organisations and NGOs, the government has implemented prevention, treatment, and rehabilitation initiatives. These efforts include increasing access to maternal healthcare, promoting family planning, and establishing fistula repair centres [13]. Additionally, community-based programs have been initiated to raise awareness about obstetric fistula and reduce stigma [14]. However, significant challenges persist in combating this issue. Limited resources, inadequate infrastructure, and geographical barriers hinder the effective delivery of healthcare services, particularly in rural areas [15]. Moreover, cultural and socio-economic factors contribute to the prevalence of early marriage, teenage pregnancy, and obstructed labour, underlying causes of obstetric fistula [13]. Overcoming these challenges requires a multi-faceted approach involving increased investment in maternal healthcare, strengthening health systems, and empowering women and girls through education and economic opportunities [16].

A comprehensive literature review reveals that while obstetric fistula has been studied extensively in sub-Saharan Africa [2, 6–12, 17–22], gaps remain in understanding the specific factors contributing to its knowledge and symptoms in Sierra Leone. Existing studies in Sierra Leone have primarily focused on understanding the effect of becoming a fistula advocate [14], the experiences of patients and medical personnel [15] and providing care for girls and women with the condition [16], often overlooking the broader context of reproductive health, including the role of education, economic status, and community-level factors. This study aims to address these gaps by assessing the knowledge of fistula symptoms among women who had a fistula and its associated factors in Sierra Leone. By doing so, the findings from this study will contribute to a more nuanced understanding of the problem, informing the development of targeted prevention and intervention strategies. Ultimately, this research seeks to contribute to the broader body of knowledge on obstetric fistula and inform policy and program development to eliminate this preventable condition.

## Materials and methods

### Study design and sampling methods

We utilised the cross-sectional data of the 2019 Sierra Leone Demographic and Health Surveys (SLDHS) for this study. The SLDHS are periodically cross-sectional surveys used to gather data on demographic, health, and nutritional factors among non-elderly adults and children. The 2019 survey was conducted over four months (from May 2019 to August 2019) [23], using a stratified, two-stage cluster sampling design. The first stage involved selecting 578 enumeration areas (EAs) from 214 urban and 364 rural regions and selecting 24 households from each EAs. This resulted in a sample size of 13,872 households [23]. The SLDHS employed a standardised, structured questionnaire administered in face-to-face interviews by trained enumerators. Rigorous quality control measures were implemented during data collection, including interviewer training, supervision, and cleaning, to ensure data accuracy and reliability. Detailed information regarding the sampling technique can be found in the final DHS report [23]. Our study comprised all 15,574 reproductive-aged women between the ages of 15 and 49 from the 2019 SLDHS. We got formal authorisation to view the entire SLDHS database by following the prescribed procedures outlined on the official DHS program website [24]. This study adhered to the Strengthening Reporting of Observational Studies in Epidemiology (STROBE) guidelines [25].

### Variables

#### Dependent variable

The dependent variable of our study was knowledge of fistula symptoms among women who had a fistula. The variables were derived based on the DHS 8 online statistics on how to calculate the knowledge of symptoms of obstetric fistula experiences from the questions in the DHS, "Ever heard of fistula and Ever experienced constant leakage of urine or stool from the vagina". Responses to this question were categorised into "no" and "yes". To derive the variable called "knowledge of fistula symptoms among women who had a fistula", we created a composite variable by combining the two variables: heard of fistula and experienced constant leakage of urine or stool from the vagina at the time of the survey. Please see the link to the DHS 8 online statistics https://dhsprogram.com/data/Guide-to-DHS-Statistics/index.htm#t=Knowledge_and_Symptoms_of_Obstetric_Fistula.htm.

#### Independent variables

The study included eighteen independent variables. These variables were grouped into individual and contextual level variables. The individual level variables include namely age (15–19, 20–24, 25–29, 30–34, 35–39, 40–49), educational level (no education, primary, secondary & higher), current working status (yes, no), religion (Christian, Islam, others), marital status (never in union, married/cohabiting, previously in a union), read newspapers or magazines (yes or no), listen to the radio (yes or no), watch television (yes or no), use the Internet (yes or no), parity (no birth, one birth, two births, three births, four or more), ever had sex (yes or no), currently pregnant (yes or no), ever terminated pregnancy (yes or no), and household size (less than 5, greater than 6). The contextual level variables include wealth index (poorest, poorer, middle, richer, richest), residence (urban, rural), household head (male or female), and region (eastern, northern, northwestern, southern, western). These variables were selected based on their significant association and previous identification as predictors of awareness of OBF [7–10].

#### Data analysis

Data was analysed in three steps using Stata version 18.0 (Stata Corporation, College Station, TX, USA). In the initial phase, we did descriptive statistics of the study population in Sierra Leone. Subsequently, a bivariate analysis was conducted to ascertain the distribution of knowledge of fistula symptoms among women who had a fistula across the explanatory variables. The findings were presented using percentages with their corresponding confidence intervals (CIs) and a p-value from a chi-square independence test to indicate their significance level. The significant variables were included in a mixed-effect multilevel binary logistic regression analysis using four models. Model I, which did not include any explanatory variables, revealed the changes in knowledge of fistula symptoms among women who had a fistula ascribed to the clustering at the primary sampling unit (PSU). In Model II, the individual level variables were included, while in Model III, the contextual level variables were included. Model IV included all the explanatory variables. The mixed-effect regression analysis yielded results that included both fixed effects and random effects. The fixed-effect results revealed the correlation between the explanatory variables and knowledge of fistula symptoms among women who had a fistula. The results were reported as an adjusted odds ratio (aOR) and their corresponding 95% confidence intervals (CI). The random effect results, however, indicate the variations in knowledge of fistula symptoms among women who had a fistula. All four models used the intra-cluster correlation coefficient (ICC) values to determine the variation. The results of the last model, which had all the explanatory variables, are reported in this paper. Before generating the results, we consistently used the weighting and the svyset function in Stata throughout the analysis.

### Ethics approval statement

The study did not seek ethical clearance for the DHS dataset, as it was publicly available. The dataset was obtained from MEASURE DHS after registration and approval, and all ethical guidelines regarding secondary dataset usage were strictly followed. More information about DHS data usage and ethical standards can be found at http://goo.gl/ny8T6X.

## Results

### Background characteristics of women in Sierra Leone

Table 1 shows the background characteristics of the women in Sierra Leone. The study population consisted primarily of adolescents in Sierra Leone, with 22.00% falling between the ages of 15–19. Education levels were low, with nearly half (45.47%) having no formal education. Despite this, a high proportion of women (68.98%) were employed. Most (62.38%) were married or cohabiting, and internet access was limited (85.61% never used it). In terms of sexual and reproductive health, a significant portion (89.73%) reported having had sex. Family sizes were generally large, with 60.08% having six or more members. Socioeconomic factors revealed a concentration of women in the richest category (23.54%) and residing in rural areas (54.00%).

**Table 1:** Background characteristics of women in Sierra Leone (n = 15574).

Variable	Weighted sample(n)	Weighted frequency (%)
**Woman’s age (years)**		
15–19	3,427	22.00
20–24	2,629	16.88
25–29	2,728	17.51
30–34	1,942	12.47
35–39	2,224	14.28
40–44	1,337	8.58
45–49	1,286	8.27
**Educational attainment**		
No education	7,081	45.47
Primary	2,103	13.50
Secondary	5,724	36.75
Higher	666	4.28
**Current working status**		
No	4,831	31.02
Yes	10,743	68.98
**Marital status**		
Never in union	5,058	32.47
Married/cohabiting	9,715	62.38
Previously married	801	5.15
**Read newspapers or magazines**		
No	14,330	92.01
Yes	1,244	7.99
**Listen to radio**		
No	8,653	55.56
Yes	6,921	44.44
**Watch television**		
No	11,143	71.55
Yes	4,431	28.45
**Use Internet**		
No	13,333	85.61
Yes	2,241	14.39
**Parity**		
No birth	4,117	26.43
One birth	2,480	15.92
Two births	2,215	14.22
Three births	1,912	12.28
Four or more births	4,850	31.15
**Ever had sex**		
No	1,599	10.27
Yes	13,975	89.73
**Currently pregnant**		
No	14,609	93.80
Yes	965	6.20
**Ever terminated pregnancy**		
No	14,246	91.47
Yes	1,328	8.53
**Religion**		
Christians	3,616	23.22
Muslims	11,953	76.75
Others	6	0.04
**Household size**		
Five and below	6,217	39.92
Six or more	9,357	60.08
**Sex of household head**		
Male	10,930	70.18
Female	4,644	29.82
**Wealth index**		
Poorest	2,738	17.58
Poorer	2,831	18.18
Middle	2,954	18.96
Richer	3,385	21.74
Richest	3,666	23.54
**Place of residence**		
Urban	7,163	46.00
Rural	8,411	54.00
**Region**		
Eastern	3,069	19.71
Northern	3,316	21.30
Northwestern	2,508	16.10
Southern	2,900	18.62
Western	3,780	24.27

Table 2 shows the bivariate analysis of knowledge of fistula symptoms among women who had a fistula in Sierra Leone. The national proportion of knowledge of fistula symptoms among women who had a fistula was 57.5% [55.3,59.7] in Sierra Leone. knowledge of fistula symptoms among women who had a fistula was highest among women aged 40–44 years 69.8%, women with higher education 73.0%, women who were working 62.0%, women who were previously married 71.1%, women with four or more births 66.2%, women who have had sex 60.9%, women were currently pregnant 62.0%, women who were Christians 60.0%, women with females as household heads 60.5%, and women living in the Southern region 60.5%. Except for watching television, wealth index and place of residence, the remaining explanatory variables showed significant associations with knowledge of fistula symptoms among women who had a fistula in Sierra Leone at p<0.05.

**Table 2:** Bivariate analysis of knowledge of fistula symptoms among women who had a fistula in Sierra Leone.

Variables	Knowledge of fistula symptoms among women who had a fistula	p-value
**Proportion**	57.5% [55.3,59.7]	
**Woman’s age (years)**		<0.001
15–19	36.8 [34.2,39.6]	
20–24	55.6 [52.4,58.7]	
25–29	62.2 [59.1,65.2]	
30–34	65.5 [62.3,68.5]	
35–39	67.1 [63.9,70.1]	
40–44	69.8 [66.2,73.1]	
45–49	65.5 [61.4,69.5]	
**Educational attainment**		<0.001
No education	59.5 [56.9,62.1]	
Primary	55.4 [51.3,59.4]	
Secondary	54.1 [51.5,56.6]	
Higher	73.0 [67.3,78.1]	
**Current working status**		<0.001
No	47.6 [44.9,50.3]	
Yes	62.0 [59.4,64.5]	
**Marital status**		<0.001
Never in union	44.6 [42.1,47.2]	
Married/cohabiting	63.1 [60.6,65.5]	
Previously married	71.1 [66.1,75.6]	
**Read newspapers or magazines**		0.026
No	57.1 [54.8,59.3]	
Yes	62.7 [57.8,67.4]	
**Listen to radio**		<0.001
No	52.1 [49.6,54.6]	
Yes	64.2 [61.6,66.8]	
**Watch television**		0.366
No	57.0 [54.6,59.5]	
Yes	58.8 [55.2,62.2]	
**Use Internet**		<0.001
No	56.2 [53.9,58.5]	
Yes	65.2 [61.0,69.3]	
**Parity**		<0.001
No birth	40.2 [37.6,42.8]	
One birth	58.6 [55.5,61.6]	
Two births	63.3 [59.6,66.9]	
Three births	64.8 [61.4,68.1]	
Four or more births	66.2 [63.5,68.8]	
**Ever had sex**		<0.001
No	27.7 [24.6,31.0]	
Yes	60.9 [58.6,63.2]	
**Currently pregnant**		<0.001
No	57.2 [55.0,59.5]	
Yes	62.0 [58.1,65.8]	
**Ever terminated pregnancy**		<0.001
No	56.3 [54.1,58.5]	
Yes	70.5 [66.0,74.6]	
**Religion**		0.040
Christians	60.0 [57.0,62.9]	
Muslims	56.8 [54.4,59.2]	
Others	36.1 [9.4,75.5]	
**Household size**		<0.001
Five and below	62.4 [59.7,65.0]	
Six or more	54.3 [52.0,56.6]	
**Sex of household head**		<0.001
Male	56.3 [53.9,58.6]	
Female	60.5 [57.7,63.2]	
**Wealth index**		0.502
Poorest	60.2 [56.0,64.3]	
Poorer	55.9 [52.6,59.2]	
Middle	57.8 [54.4,61.1]	
Richer	57.3 [53.1,61.5]	
Richest	56.7 [52.9,60.4]	
**Place of residence**		0.496
Urban	56.7 [53.3,60.0]	
Rural	58.2 [55.3,61.1]	
**Region**		0.042
Eastern	60.4 [55.3,65.3]	
Northern	59.2 [54.3,64.0]	
Northwestern	56.8 [52.8,60.7]	
Southern	60.5 [56.2,64.7]	
Western	51.9 [46.8,57.0]	

*P-values were generated from a Chi-square test

### Factors associated with knowledge of fistula symptoms among women who had a fistula in Sierra Leone

#### 1. Socio-demographics

Women aged 20–49 had higher odds of knowledge of fistula symptoms among women who had a fistula compared to those aged 15–19, with the highest odds among those aged 40–44 (aOR = 2.82; 95% CI: 2.13, 3.73). Women with primary, secondary, and higher education had increased odds of knowledge of fistula symptoms among women who had a fistula compared to those without education. The highest odds were observed among women with higher education (aOR = 2.07; 95% CI: 1.49, 2.88). Women who were working had higher odds of knowledge of fistula symptoms among women who had a fistula than those who were not working (aOR = 1.33; 95% CI: 1.14, 1.56). Women with female household heads had increased odds of knowledge of fistula symptoms among women who had a fistula compared to those with male household heads (aOR = 1.20; 95% CI: 1.05, 1.38). Women living in larger households (six or more members) had lower odds of knowledge of fistula symptoms among women who had a fistula compared to those in smaller households (five or below) (aOR = 0.86; 95% CI: 0.75, 0.97). The odds of knowledge of fistula symptoms among women who had a fistula were lower among women living in the Western region compared to those in the Eastern region (aOR = 0.48; 95% CI: 0.31, 0.75).

#### 2. Sexual history

Women who had engaged in sexual activity had higher odds of knowledge of fistula symptoms among women who had a fistula compared to those who had not (aOR = 2.19; 95% CI: 1.73, 2.77). Pregnant women had higher odds of knowledge of fistula symptoms among women who had a fistula than those who were not pregnant (aOR = 1.37; 95% CI: 1.13, 1.66). Women who had ever terminated a pregnancy had higher odds of knowledge of fistula symptoms among women who had a fistula compared to those who had not (aOR = 1.30; 95% CI: 1.07, 1.59).

#### 3. Obstetric history

Women with one, two, three, or four or more births had higher odds of knowledge of fistula symptoms among women who had a fistula compared to those without any births. The highest odds were among those with four or more births (aOR = 2.00; 95% CI: 1.57, 2.54).

#### 4. Media exposure

Women who listened to the radio had higher odds of knowledge of fistula symptoms among women who had a fistula than those who did not (aOR = 1.47; 95% CI: 1.30, 1.67). Women who used the internet had higher odds of knowledge of fistula symptoms among women who had a fistula compared to those who did not (aOR = 1.64; 95% CI: 1.32, 2.05).

### Random effects results

Table 3 indicates variations in the factors associated with knowledge of fistula symptoms among women who had a fistula in Sierra Leone among the clusters (σ2 = 2.25, 95% CI = 1.91 to 2.64) in model I. Approximately 40% of the proportion of knowledge of fistula symptoms among women who had a fistula was attributed to the variations between the clusters (intraclass correlation (ICC) = 0.40). The between-cluster difference increased to 44% in Model II, decreased to 40% in Model III, and increased to 44% in Model IV. These ICC results suggest that the likelihood of knowledge of fistula symptoms among women who had a fistula variations can be attributed to the variances across the clusters.

**Table 3:** Factors associated with knowledge of fistula symptoms among women who had a fistula in Sierra Leone.

Variables	Model I Empty model	Model II aOR [95% CI]	Model III aOR [95% CI]	Model IV aOR [95% CI]
**Fixed effect results**				
**Women’s age(years)**				
15–19		1.00		1.00
20–24		1.51*** [1.26,1.80]		1.50*** [1.26,1.80]
25–29		1.87*** [1.51,2.30]		1.86*** [1.51,2.30]
30–34		2.32*** [1.79,3.01]		2.31*** [1.78,3.00]
35–39		2.43*** [1.86,3.18]		2.41*** [1.84,3.16]
40–44		2.86*** [2.16,3.77]		2.82*** [2.13,3.73]
45–49		2.45*** [1.83,3.27]		2.41*** [1.80,3.22]
**Educational attainment**				
No education		1.00		1.00
Primary		1.22* [1.03,1.45]		1.22* [1.03,1.44]
Secondary		1.51*** [1.30,1.75]		1.51*** [1.30,1.76]
Higher		2.06*** [1.48,2.86]		2.07*** [1.49,2.88]
**Current working status**				
Not working		1.00		1.00
Working		1.34*** [1.15,1.56]		1.33*** [1.14,1.56]
**Marital status**				
Never in union		1.00		1.00
Married/cohabiting		0.95 [0.81,1.11]		1.00 [0.85,1.17]
Previously married		1.19 [0.91,1.56]		1.16 [0.88,1.52]
**Read newspapers or magazines**				
No		1.00		1.00
Yes		1.12 [0.90,1.39]		1.13 [0.91,1.40]
**Listen to radio**				
No		1.00		1.00
Yes		1.46*** [1.29,1.66]		1.47*** [1.30,1.67]
**Use Internet**				
No		1.00		1.00
Yes		1.65*** [1.32,2.06]		1.64*** [1.32,2.05]
**Parity**				
No birth		1.00		1.00
One birth		1.59*** [1.35,1.87]		1.56*** [1.33,1.84]
Two births		1.75*** [1.39,2.21]		1.73*** [1.38,2.18]
Three births		1.90*** [1.51,2.39]		1.87*** [1.49,2.36]
Four or more births		2.03*** [1.60,2.58]		2.00*** [1.57,2.54]
**Ever had sex**				
No		1.00		1.00
Yes		2.20*** [1.74,2.77]		2.19*** [1.73,2.77]
**Currently pregnant**				
No		1.00		1.00
Yes		1.37** [1.13,1.66]		1.37** [1.13,1.66]
**Ever terminated pregnancy**				
No		1.00		1.00
Yes		1.30** [1.07,1.59]		1.30** [1.07,1.59]
**Religion**				
Christians		1.00		1.00
Muslims		1.03 [0.90,1.19]		1.04 [0.90,1.19]
Others		0.53 [0.03,8.34]		0.51 [0.03,8.77]
**Household size**				
Five and below		1.00		1.00
Six or more		0.84** [0.74,0.95]		0.86* [0.75,0.97]
**Sex of household head**				
Male			1.00	1.00
Female			1.15* [1.03,1.30]	1.20** [1.05,1.38]
**Region**				
Eastern			1.00	1.00
Northern			0.87 [0.59,1.29]	0.82 [0.51,1.31]
Northwestern			0.81 [0.58,1.14]	0.83 [0.53,1.28]
Southern			1.02 [0.70,1.49]	1.02 [0.67,1.56]
Western			0.62** [0.43,0.89]	0.48** [0.31,0.75]
**Random effect model**				
PSU variance (95% CI)	2.25 [1.91, 2.64]	2.67 [2.28, 3.12]	2.22 [1.89, 2.60]	2.59 [2.22, 3.03]
ICC	0.40 [0.36, 0.44]	0.44 [0.40, 0.48]	0.40 [0.36, 0.44]	0.44 [0.40, 0.47]
N	15574	15574	15574	15574
Number of clusters	576	576	576	576

aOR = adjusted odds ratios; CI = Confidence Interval

* p<; 0.05

** p<; 0.01

*** p<; 0.001; 1.00 = Reference category; PSU = Primary Sampling Unit; ICC = Intra-Class Correlation Coefficient

## Discussion

Our study assessed the knowledge of fistula symptoms among women who had a fistula in Sierra Leone, finding that 57.5% were aware of these symptoms. Awareness was higher among women aged 20–49, especially those aged 40–44, and those with higher education levels. Factors associated with increased knowledge included being employed, listening to the radio, using the internet, having multiple births, and having sexual experience, being pregnant, or having terminated a pregnancy. Additionally, having a female household head was linked to greater awareness, while larger household sizes and residing in the Western region were associated with lower knowledge levels.

Our study revealed that 57% of women in Sierra Leone had knowledge of fistula symptoms among women who had a fistula. This is higher than what was reported in other sub-Saharan African countries like Ghana [26], Ethiopia [27], and Nigeria [28]. Variations in data collection methods, sample sizes, and how knowledge of fistula symptoms among women who had a fistula were assessed in our study compared to the other studies account for the differences. The possible reason for the findings in our research in Sierra Leone may be due to the UNFPA’s Global Campaign to End Fistula in Sierra Leone, which aims to improve access to quality maternal health services and raise awareness of sexual and reproductive health and rights [13]. The activities conducted by UNFPA include leaflets, posters, community dialogue meetings, radio jingles, and discussions with fistula survivors as champions of fistula prevention [13]. Despite the work that has been done, almost half of the women were not aware of OBF. This means more work needs to be done by sexual and reproductive health organisations to intensify their health promotion efforts, especially targeting women with specific characteristics that this study has identified as potential risk factors.

Women aged 20–49, particularly those between 40–44, were more likely to be aware of the knowledge of fistula symptoms among women who had a fistulacompared to teenagers (15–19). This finding aligns with the previous studies in Ethiopia [10] and Uganda [29]. Older women in Sierra Leone are more aware of fistula due to their life experience, exposure to health information, and involvement in community activities [13, 30]. They may have witnessed or heard about fistula cases in their communities, learned about its causes and prevention, and had more access to media disseminating health messages [13, 30]. They may also have more decision-making power in their households, allowing them to seek healthcare or support when needed [8, 9].

Women with higher education levels (primary, secondary, or higher) had knowledge of fistula symptoms among women who had a fistula compared to those without education, highlighting the significant impact of education on awareness in Sierra Leone. This aligns with previous studies [10, 20, 27] indicating that educated women are more likely to be informed about obstetric fistula due to better access to information through various media and healthcare services [31]. Educated women are also more aware of their health rights and empowered to challenge harmful practices like female genital mutilation, early marriage, and gender-based violence [27]. Additionally, working women who engaged with media, such as listening to the radio or using the internet, showed increased knowledge of fistula symptoms among women who had a fistula. This reflects findings from other sub-Saharan African studies [8, 10, 32], suggesting that employment broadens social networks and enhances financial autonomy, facilitating access to healthcare and information. Media exposure further empowers women by increasing knowledge about fistula and encouraging them to seek appropriate medical care [16, 33].

The study reveals that women with lifetime births were more likely to know the knowledge of fistula symptoms among women who had a fistula. The result contradicts Asefa et al. [27] and the previous study in sub-Saharan Africa [8]. In those studies, parity was less associated with OBF. Fistula is caused by prolonged labour, and women with more children are more likely to have experienced or witnessed this complication and may be aware of it [34]. Fistula prevention and treatment programs target women with more children, increasing awareness of potential risks and complications [13].

The study found an association between pregnancy termination and knowledge of fistula symptoms among women who had a fistula. Aleminew et al. [10], Tweneboah et al. [9], and Asefa et al. [27] reported similar findings. Women who terminated their pregnancies likely experienced complications or risks that increased their awareness of reproductive health complications, such as the risk of fistula [8]. They may have received more information or counselling from healthcare providers or organisations offering safe abortion services. The study also revealed that pregnant women are more likely to be aware of obstetric fistula. Pregnancy often leads to more frequent visits to healthcare facilities for antenatal care, increasing the chances of exposure to information about obstetric fistula. The study also reveals that women who have had sex were more likely to be aware of OBF. Our finding is inconsistent with the previous study in sub-Saharan Africa [8]. In that study, women who had never had sex were less likely to be aware of OBF. Women who have had sexual or reproductive experiences are more likely to seek healthcare, increasing their chances of learning about obstetric fistula.

Our findings revealed that larger household sizes and living in the Western region were associated with a lower likelihood of knowledge of fistula symptoms among women who had a fistula. Larger households often face economic challenges, limiting healthcare, education, and information access. Cultural norms and beliefs about women’s health and roles might differ between regions. Further research is needed to understand these associations fully.

### Policy and practice implications

Our study provides crucial insights for policy development to address obstetric fistula in Sierra Leone. Given the disparities in knowledge across age groups, educational campaigns should be tailored to specific age groups. For instance, comprehensive sex education for adolescents and reproductive health information for women of childbearing age is essential. Efforts should prioritise women with low education, those in rural areas, and those from specific ethnic groups, as these groups are particularly vulnerable to a lack of knowledge about obstetric fistula. Investment in maternal healthcare, including access to skilled birth attendants, is crucial to prevent obstetric fistula. Leveraging radio and the internet to disseminate information about obstetric fistula is essential. Policies that empower women, such as promoting education and economic opportunities, can contribute to increased knowledge and access to healthcare. Healthcare providers and community health workers should conduct outreach programs to raise awareness about obstetric fistula, focusing on vulnerable groups. Integration of obstetric fistula prevention and management into routine maternal healthcare services is crucial. Training healthcare providers on preventing, diagnosing, and managing obstetric fistula is essential. Partnerships between healthcare providers, community-based organisations, and media outlets can enhance the impact of interventions. Regular monitoring and evaluation of interventions are necessary to assess their effectiveness and make necessary adjustments. By addressing these policy and practice implications, Sierra Leone can make significant progress in reducing the prevalence of obstetric fistula and improving the lives of affected women.

### Strengths and limitations

Using a nationally representative dataset, this study is the first to assess the knowledge of fistula symptoms among women who had a fistula, and factors associated with it in Sierra Leone. This is a meaningful contribution to existing literature. However, the cross-sectional nature of secondary data limits causal inferences. Additionally, the DHS does not disaggregate questions to show specific types of fistulae (i.e. vesicovaginal fistula, urethrovaginal fistula, or rectovaginal fistula) that women know. Recall bias was another limitation of the study. Another key limitation of this study was the unavailability of detailed reproductive health data within the 2019 Sierra Leone Demographic and Health Survey dataset. Consequently, crucial variables such as puberty, menarche, menopause, and specific health conditions that could significantly influence obstetric fistula knowledge and symptoms were not included in the analysis. This limitation restricts the depth of our investigation into the complex factors associated with obstetric fistula awareness. Future studies with more comprehensive data on reproductive health will be essential further to elucidate the predictors of obstetric fistula knowledge and symptoms.

## Conclusion

Most reproductive-aged women in Sierra Leone have knowledge of fistula symptoms among women who had a fistula. Factors such as age, education, occupation, media exposure, parity, sexual activity, pregnancy status, abortion history, ethnicity, household structure, and geographic location influence the knowledge of fistula symptoms among women who had a fistula. Based on these findings, the government and partner organisations in Sierra Leone should implement comprehensive health education programs targeting reproductive-aged women, with a specific focus on obstetric fistula prevention, symptoms, and available treatment options. Promote media campaigns to raise awareness about obstetric fistula, using accessible language and culturally appropriate messages. Expand access to education for girls and women, especially in rural areas, to empower them with knowledge about reproductive health. Enhance maternal healthcare services, including access to skilled birth attendants, to reduce the risk of obstetric fistula. Collaborate with community leaders to address cultural and social norms that contribute to obstetric fistula, such as early marriage and harmful traditional practices.

## Abbreviations

aOR: Adjusted Odds Ratio
CI: Confidence Intervals
DHS: Demographic Health Survey
OBF: Obstetric Fistula
ICC: Intraclass Correlation
SLDHS: Sierra Leone Demographic and Health Surveys
